# Seronegative Autoimmune Encephalitis: A Case Report and Literature Review on Psychiatric Presentations

**DOI:** 10.1155/crps/9618597

**Published:** 2026-07-26

**Authors:** Sercan Karabulut, Hüseyin Oğuz Uyaroğlu, Ebru Barçın

**Affiliations:** ^1^ Psychiatry Clinic, Akdeniz University Hospital, Akdeniz University, Antalya, Türkiye, akdeniz.edu.tr; ^2^ Neurology Clinic, Akdeniz University Hospital, Akdeniz University, Antalya, Türkiye, akdeniz.edu.tr

**Keywords:** autoimmune encephalitis, case report, psychiatry, seronegative

## Abstract

Autoimmune encephalitis (AE) is an immune‐mediated disorder that may present with prominent psychiatric symptoms, posing diagnostic challenges, particularly in seronegative cases. We report a 57‐year‐old male presenting with acute behavioral changes, psychosis, and cognitive impairment, initially managed in a psychiatric setting without clinical improvement. Subsequent evaluation revealed EEG abnormalities and elevated cerebrospinal fluid (CSF) protein, while autoimmune antibody panels were negative, leading to a diagnosis of seronegative probable AE. The patient showed marked clinical improvement following immunotherapy. This case highlights the importance of considering AE in patients with atypical psychiatric presentations, particularly when accompanied by cognitive decline or neurological findings. At the same time, careful clinical judgment is essential, as overdiagnosis and indiscriminate testing may lead to unnecessary interventions. Early recognition, guided by clinical red flags and supported by objective findings, remains critical for optimal outcomes.

## 1. Introduction

Autoimmune encephalitis (AE) encompasses a group of immune‐mediated inflammatory disorders that affect the brain parenchyma in the absence of infectious causes. These conditions commonly involve the cortical or deep gray matter and may also extend to the white matter, meninges, or spinal cord [1]. Clinically, AE presents with a broad spectrum of symptoms including behavioral disturbances, psychotic features, seizures, cognitive and memory impairments, abnormal movements, dysautonomia, and decreased levels of consciousness [2]. Given the risk that AE may be overlooked in patients presenting with predominantly psychiatric symptoms, the presence of features such as memory deficits, seizures, dyskinesias, and unexplained headaches should prompt heightened clinical suspicion [3].

AE includes a range of syndromes with varying pathophysiological mechanisms. One subgroup comprises classical paraneoplastic syndromes associated with antibodies against intracellular neuronal antigens, such as anti‐Hu, with pathology predominantly mediated by T‐cell responses targeting neuronal elements. Another subgroup involves antibodies directed against extracellular domains of ion channels, receptors, and related proteins—examples include antibodies targeting the NMDA receptor [4]. Additionally, a third category—termed seronegative probable AE—describes cases where no known autoantibodies are detected in cerebrospinal fluid (CSF) or serum [5]. It is proposed that these cases may be attributable to yet‐undiscovered antibodies [6], which complicate timely diagnosis and treatment.

With growing awareness of AE’s diverse clinical manifestations and the rising recognition of seronegative AE as a major subtype [7], this case report aims to illustrate the diagnostic and therapeutic challenges associated with seronegative AE.

The authors obtained informed consent from the patient prior to the publication. Additional supporting information can be found online in the Supporting Information section.

## 2. Case Presentation

A 57‐year‐old male with no significant medical history presented to the psychiatric outpatient clinic with behavioral changes and acute alterations in the mental status. Over the preceding month, he exhibited agitation, confabulation, paranoid delusions, and visual hallucinations. Cognitive dysfunction was evident, as he was unable to recognize his spouse or orient himself to his home environment. On neurological examination, no focal motor or sensory deficit was detected. There were no meningeal signs or abnormal involuntary movements. The patient showed fluctuating orientation, impaired short‐term memory, confabulation, paranoid delusions, visual hallucinations, and behavioral dysregulation. Formal neuropsychological testing could not be performed during the acute phase because of agitation and fluctuating cooperation.

A detailed history revealed a prodromal episode 6 weeks earlier characterized by fatigue, anergia, and appetite reduction, preceding the onset of neuropsychiatric symptoms. Subsequently, his orientation fluctuated and his sleep–wake cycle became disrupted. He exhibited short‐term memory loss and failed to recognize family members, displaying aggression toward his own spouse. Paranoid delusions, particularly the fear of poisoning, led to avoidant behaviors and further appetite decline.

**Figure 1:**
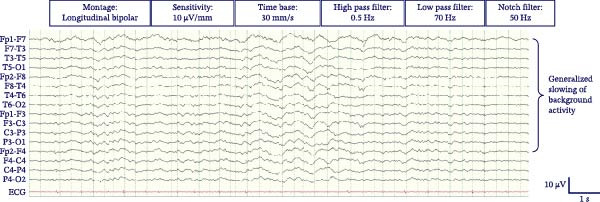
Electroencephalogram demonstrating generalized slowing of background activity. Recording was obtained using a longitudinal bipolar montage (sensitivity: 10 μV/mm and time base: 30 mm/s).

Four weeks prior to the psychiatric outpatient admission, he presented to the emergency department. Brain MRI findings were unremarkable. EEG demonstrated generalized slowing (Figure 1). Lumbar puncture revealed elevated CSF protein (135 mg/dL). Because of prominent psychiatric symptoms, namely, agitation, confabulations, paranoid delusions, and visual hallucinations, psychiatric treatment was prioritized, and the patient was transferred to the inpatient psychiatry service of another hospital due to the lack of bed availability at our center. Differential diagnosis included major depressive disorder. Haloperidol (2 mg/day) and diazepam (5 mg/day) were initiated to manage agitation, while escitalopram (10 mg/day) was added for depressive symptoms accompanied by psychotic features. Despite 5 days of hospitalization, no clinical improvement was observed.

**Figure 2:**
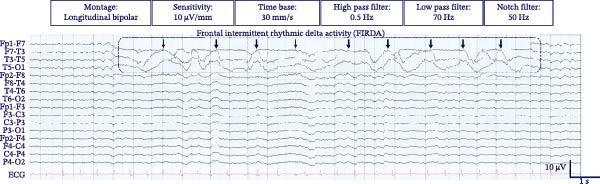
Electroencephalogram showing frontal intermittent rhythmic delta activity (FIRDA) indicated by arrows. Recording was obtained using a longitudinal bipolar montage (sensitivity: 10 μV/mm and time base: 30 mm/s).

Following psychiatric evaluation, the patient was transferred to the neurology inpatient unit of our center with a preliminary diagnosis of probable AE. Psychiatric medications were discontinued, and haloperidol was replaced with olanzapine (5 mg/day) due to an inefficient response to haloperidol treatment. Laboratory evaluations, including complete blood count, renal and hepatic panels, and electrolyte levels, were all within normal limits. EEG was repeated and revealed frontal intermittent rhythmic delta activity (Figure 2). A second MRI again showed no pathological findings. Repeat lumbar puncture demonstrated elevated protein (80 mg/dL), normal glucose, and a white blood cell count (4 cells/µL). Infectious workup—including brucellosis, HSV, borreliosis, EBV, CMV, and tuberculosis—was negative. No oligoclonal bands were detected; the IgG index was negative, and CSF cytology was nondiagnostic. Serum Ca 12‐5, Ca 15‐3, Ca 19‐9, and Ca 72‐4 levels were within the normal range. CT scans of the chest, abdomen, and pelvis revealed no malignancy.

Autoimmune panel results were negative for anti‐NMDAR, AMPAR, Caspr2, LGI1, GABAR, and DPPX antibodies. The paraneoplastic antibody panel was not performed due to technical limitations. Based on the clinical picture, EEG abnormalities, and lack of serological markers, a diagnosis of seronegative probable AE was established. The patient received 12 days of intravenous methylprednisolone (1 g/day). His cognitive symptoms and memory improved; however, agitation and psychosis persisted. Due to persistent symptoms and EEG findings, the olanzapine dose was increased to 10 mg/day, and carbamazepine (200 mg/day) was added. Clinical improvement was subsequently observed, and the patient returned to baseline mental status by day 25 of hospitalization. A follow‐up EEG confirmed the resolution of abnormalities. During inpatient treatment, medication administration was supervised by the clinical team. No major adverse events related to intravenous methylprednisolone, olanzapine, or carbamazepine were documented. The patient tolerated the treatment regimen without any discontinuation due to side effects. At a 4‐month follow‐up, he remained clinically asymptomatic, and no relapse was reported.

After clinical recovery, the patient was invited to share his perspective on the illness and treatment process. He reported limited memory of the acute phase but described the overall experience as distressing for both himself and his family. He expressed satisfaction with the multidisciplinary evaluation and clinical improvement after treatment. Table 1 shows the timeline of the clinical course.

**Table 1:** Timeline of the clinical course.

Time point	Clinical event/findings	Diagnostic or therapeutic action
Six weeks before psychiatric outpatient admission	Fatigue, anergia, appetite reduction	No specific treatment reported
Approximately 1 month before admission	Behavioral changes, agitation, paranoid delusions, visual hallucinations, confabulation, fluctuating orientation, short‐term memory impairment	Initial psychiatric evaluation considered
Four weeks before psychiatric outpatient admission	Emergency department presentation; MRI unremarkable; EEG showed generalized slowing; CSF protein 135 mg/dL	Psychiatric hospitalization due to predominant psychiatric symptoms and bed unavailability at the authors’ center
Psychiatric hospitalization, 5 days	Persistent agitation, psychosis, and cognitive impairment; no clinical improvement	Haloperidol 2 mg/day, diazepam 5 mg/day, escitalopram 10 mg/day
Transfer to neurology inpatient unit	Preliminary diagnosis of probable/suspected AE	Psychiatric medications discontinued; olanzapine 5 mg/day initiated
Neurology admission workup	Repeat EEG showed FIRDA; repeat MRI unremarkable; repeat CSF protein 80 mg/dL, WBC 4 cells/µL; infectious workup negative; autoimmune panel negative	Diagnosis considered as suspected seronegative autoimmune encephalitis
Treatment period	Persistent cognitive and psychotic symptoms initially	Intravenous methylprednisolone 1 g/day for 12 days; olanzapine increased to 10 mg/day; carbamazepine 200 mg/day added
Day 25 of hospitalization	Return to baseline mental status	Follow‐up EEG showed resolution of abnormalities
Four‐month follow‐up	Patient remained asymptomatic	Clinical follow‐up continued

## 3. Discussion

The concept of AE dates back to the 1960s, when the condition was described as “limbic encephalitis” in patients with acute inflammatory involvement of limbic structures, marked by memory deficits, seizures, and psychiatric symptoms ([8]). The diagnostic framework evolved through the 1980s with the identification of paraneoplastic associations. However, the recognition of nonparaneoplastic forms and the discovery of antibodies targeting synaptic proteins led to the classification of AE as a distinct clinical entity [9].

According to the 2016 diagnostic criteria, a diagnosis of “possible AE” requires subacute onset (less than 3 months) of memory deficits, altered mental status, or psychiatric symptoms, along with at least one of the following: new focal CNS findings, new‐onset seizures, CSF pleocytosis, or MRI abnormalities [10]. To clarify the diagnostic level of certainty, the present case was explicitly mapped to the Graus et al. [10] diagnostic criteria. The patient met the clinical criterion of rapid/subacute progression within less than 3 months, with prominent psychiatric symptoms, altered mental status, and short‐term memory impairment. However, the case did not fulfill all strict criteria for possible AE because there were no new focal central nervous system findings, no new‐onset seizures, no CSF pleocytosis, and no MRI findings suggestive of encephalitis. Table 2 shows the mapping of the case to Graus et al. [10] criteria.

**Table 2:** Mapping of the present case to Graus et al. [10] diagnostic criteria.

Diagnostic criterion	Findings in the present case	Assessment
Subacute onset, less than 3 months, of working memory deficits, altered mental status, or psychiatric symptoms	The patient developed behavioral change, paranoid delusions, visual hallucinations, confabulation, fluctuating orientation, short‐term memory impairment, and failure to recognize family members over approximately 1 month	Met
New focal central nervous system findings	No new focal neurological deficit was reported	Not met
New‐onset seizures	No seizure history was reported	Not met
CSF pleocytosis	CSF white blood cell count was 4 cells/µL	Not met
MRI features suggestive of encephalitis	Two brain MRI examinations were unremarkable	Not met
Reasonable exclusion of alternative causes	Routine laboratory tests were within normal limits; infectious workup, including brucellosis, HSV, borreliosis, EBV, CMV, and tuberculosis, was negative; CSF cytology was nondiagnostic; tumor markers and CT scans of the chest, abdomen, and pelvis did not reveal malignancy. However, the paraneoplastic antibody panel could not be performed because of technical limitations	Partial met
Absence of well‐characterized neuronal autoantibodies	Autoimmune antibody testing was negative for anti‐NMDAR, AMPAR, Caspr2, LGI1, GABAR, and DPPX antibodies	Met
Supportive criteria for autoantibody‐negative but probable autoimmune encephalitis: MRI abnormalities, CSF inflammatory findings, or brain biopsy	MRI was normal; CSF pleocytosis was absent; oligoclonal bands were not detected; IgG index was negative; and brain biopsy was not performed. EEG abnormalities and elevated CSF protein supported cerebral dysfunction but are nonspecific and are not sufficient alone to fulfill the formal Graus criteria for probable autoantibody‐negative autoimmune encephalitis	Not met

AE can affect individuals of all ages and genders, with either acute or subacute progression depending on the subtype. Serological and CSF screening for autoantibodies can aid in subtype differentiation and prognosis. The most commonly implicated cell‐surface antibodies include those against NMDAR, AMPAR, LGI1, Caspr2, GAD, and GABABR. Clinical features often align with the implicated antibody subtype, and removal of pathogenic antibodies via immunotherapy or plasmapheresis typically results in improvement [11, 12].

Nevertheless, nearly 50% of AE cases are seronegative, lacking identifiable antibody markers [6, 13]. As in prior reports, our case demonstrated features overlapping with antibody‐positive AE.

Psychiatric symptoms are well‐recognized features of AE and often dominate the early clinical presentation. In our case, psychiatric symptoms predominated during the early phase. Anti‐NMDAR encephalitis, for instance, frequently presents with psychiatric features in young women, with up to 80%–90% initially evaluated in psychiatric settings [14, 15].

A recent study examining long‐term outcomes in AE reported that neuropsychiatric symptoms such as fatigue, mood instability, and impaired memory and attention were common [16]. Similarly, Lee et al. [7] found high rates of psychiatric symptoms (76.9%), CSF abnormalities (71.8%), and EEG changes (83%) among patients with seronegative probable AE. Early diagnosis and treatment were associated with better long‐term recovery [17].

These presentations are often misdiagnosed as brief psychotic disorder, manic episodes, or first‐episode schizophrenia [18]. The differential diagnosis of our case was broad because the initial presentation was dominated by psychiatric and cognitive symptoms. Primary psychiatric disorders, including late‐onset psychosis, brief psychotic disorder, and major depressive disorder with psychotic features, were considered, particularly because agitation, paranoid delusions, visual hallucinations, and appetite reduction were prominent at presentation. However, the patient had no previous psychiatric history, and the subacute onset, fluctuating orientation, severe short‐term memory impairment, failure to recognize family members, sleep–wake cycle disruption, and lack of early response to antipsychotic and antidepressant treatments were atypical for a primary psychiatric disorder alone. Delirium secondary to metabolic, toxic, or systemic medical causes was also considered. This was less likely because routine laboratory investigations, including complete blood count, renal and hepatic panels, and electrolyte levels, were within normal limits, and there was no evidence of an acute systemic illness explaining the clinical picture. Infectious encephalitis was another important differential diagnosis, but the infectious workup, including testing for herpes simplex virus, brucellosis, borreliosis, Epstein–Barr virus, cytomegalovirus, and tuberculosis, was negative. Seizure‐related conditions, including nonconvulsive seizures or postictal states, were considered in view of the fluctuating mental status and EEG abnormalities; however, no clinical seizure history was reported, and the EEG findings were nonspecific rather than clearly epileptiform.

Structural, neoplastic, and paraneoplastic etiologies were also evaluated. Two brain MRI examinations did not reveal structural lesions or imaging features suggestive of encephalitis, and CT scans of the chest, abdomen, and pelvis did not demonstrate malignancy. Serum tumor markers were within the normal limits. Nevertheless, a paraneoplastic etiology could not be completely excluded because the paraneoplastic antibody panel could not be performed due to technical limitations. Rapidly progressive neurodegenerative disorders were considered less likely because of the acute/subacute time course, the presence of objective EEG and CSF abnormalities, and the marked clinical recovery after immunotherapy and supportive neuropsychiatric treatment.

In line with the Graus diagnostic criteria for AE, the diagnosis in this case was not based solely on psychiatric symptoms but on the integration of clinical and paraclinical features. The absence of movement disorders or a seizure history, which are among the commonly reported neurological manifestations of AE, should also be taken into account in the differential diagnosis. At the same time, it is important to acknowledge that overdiagnosis of AE—particularly in seronegative cases—has emerged as a clinical concern, as misattribution of primary psychiatric disorders to an autoimmune etiology may expose patients to unnecessary immunotherapies and potential iatrogenic harm [19]. Therefore, careful integration of the clinical presentation with supportive objective findings is essential, and the diagnosis should be approached with caution in the absence of clear neurological or paraclinical evidence.

Importantly, not all patients presenting with first‐episode psychosis require extensive neurological investigations, as indiscriminate testing may lead to false‐positive findings and diagnostic uncertainty. Instead, further assessment should be guided by the presence of subacute onset, rapid progression, prominent cognitive impairment, fluctuating consciousness, or accompanying neurological features.

In this context, it is also essential to recognize the limitations of paraclinical investigations. Findings such as EEG slowing or mild CSF abnormalities are often nonspecific, and a substantial proportion of patients with AE—both seropositive and seronegative—may have normal MRI, EEG, or CSF results [10, 14]. Therefore, these investigations should be interpreted within a broader clinical context rather than used in isolation.

Although high‐dose corticosteroids are usually administered for 3–5 days, in this case, the decision to extend intravenous methylprednisolone to 12 days was guided by the patient’s partial but incomplete clinical response after the initial days of treatment. Regarding second‐line immunotherapies, such as IVIg or rituximab, there were no features suggestive of a severe or refractory course that would necessitate early second‐line treatment. IVIg or rituximab may be considered in cases with insufficient response to first‐line therapy, and their use should be guided by disease severity and clinical progression. In our case, olanzapine and carbamazepine were used as adjunctive symptomatic treatments for persistent agitation, psychosis, and behavioral dysregulation rather than as etiological treatment. The management of psychiatric symptoms in AE is largely supportive and should be considered adjunctive to immunotherapy. Antipsychotic or antiepileptic medications may be used to control agitation and psychosis; however, cautious use is recommended, particularly at low doses, due to the risk of adverse effects and increased sensitivity observed in some patients [20].

Some evidence suggests that elevated dopamine levels, secondary to NMDA receptor hypofunction, may underlie psychotic features [19]. Such psychiatric presentations frequently delay appropriate diagnosis and management. Given the critical importance of early intervention, psychiatrists play a key role in recognizing atypical neuropsychiatric presentations that warrant neurological assessment.

A strength of this case report is the longitudinal description of a diagnostically challenging neuropsychiatric presentation, including repeated EEG, CSF evaluation, neuroimaging, infectious workup, malignancy screening, treatment course, and follow‐up. The report also highlights the need for interdisciplinary collaboration between psychiatry and neurology. However, several limitations should be acknowledged. This is a single‐patient report, and causal inference regarding the treatment response is limited. Formal neuropsychological testing was not available during the acute phase. The paraneoplastic antibody panel could not be performed because of technical limitations. In addition, EEG abnormalities and elevated CSF proteins are supportive but nonspecific findings. Therefore, the diagnosis should be interpreted cautiously as clinically suspected seronegative AE.

## 4. Conclusion

Psychiatrists must maintain a high index of suspicion for AE, particularly in patients with no prior psychiatric history who present with acute cognitive or behavioral changes. Neurological evaluation—including lumbar puncture, EEG, and neuroimaging—should be considered in such cases. Importantly, negative CSF antibody panels do not exclude AE. Early recognition and prompt immunotherapy can lead to full recovery even in seronegative cases, highlighting the need for interdisciplinary collaboration in the diagnosis and management.

## Funding

The authors received no specific funding for this work.

## Disclosure

An earlier version of this manuscript was posted as a public prepublication version on ResearchGate in 2025: Karabulut S, Uyaroğlu HO, Barçın E. Seronegative Autoimmune Encephalitis: A Case Report and Literature Review on Psychiatric Presentations. (https://www.researchgate.net/publication/393842348_Seronegative_Autoimmune_Encephalitis_A_Case_Report_and_Literature_Review_on_Psychiatric_Presentations). This preprint was disclosed to the journal. The present manuscript has been revised following editorial and reviewer feedback, and the preprint record will be updated with a link to the final published version after publication [21].

## Ethics Statement

This case report describes the clinical course of a single patient and does not involve any experimental intervention or research procedure beyond routine clinical care. According to the Akdeniz University Hospital Ethical Committee, formal ethics committee approval was not required for an anonymized single‐patient case report. The report was prepared in accordance with the principles of the Declaration of Helsinki, and all potentially identifying patient information was removed. This case report was prepared in accordance with the CARE 2013 reporting guidelines.

## Consent

Written informed consent was obtained from the patient for publication of this case report, including the clinical details and accompanying EEG figures, after clinical recovery and return to baseline mental status.

## Conflicts of Interest

The authors declare no conflicts of interest.

## Supporting Information

Additional supporting information can be found online in the Supporting Information section.

## Supporting information


**Supporting Information** Completed CARE checklist submitted in accordance with CARE reporting guidelines.

## Data Availability

Data sharing is not applicable to this article because no datasets were generated or analyzed during the preparation of this case report. All relevant de‐identified clinical information supporting the conclusions of this report is included in the article. Additional patient‐level data cannot be made publicly available due to the privacy and confidentiality restrictions.
