# Supplementary material for: Seronegative Autoimmune Encephalitis: A Case Report and Literature Review on Psychiatric Presentations
Source: Case Rep Psychiatry. 2026 Jul 26;2026:9618597. doi: 10.1155/crps/9618597 (PMC13402879; doi:10.1155/crps/9618597)
Supplement: Supplementary file 1 — Supporting Information Completed CARE checklist submitted in accordance with CARE reporting guidelines. [file CRPS-2026-9618597-s001.pdf]

CARE case report guidelines

CARE Checklist of information to include when writing a case report

(cc) BY-NC-ND

☑

Topic	Item	Checklist item description	Reported on Line
Title	1	The diagnosis or intervention of primary focus followed by the words "case report"	p. 1
Key Words	2	2 to 5 key words that identify diagnoses or interventions in this case report, including "case report"	p. 2
Abstract (no references)	3a	Introduction: What is unique about this case and what does it add to the scientific literature?	p. 2
	3b	Main symptoms and/or important clinical findings	p. 2
	3c	The main diagnoses, therapeutic interventions, and outcomes	p. 2
	3d	Conclusion—What is the main "take-away" lesson(s) from this case?	p. 2
Introduction	4	One or two paragraphs summarizing why this case is unique (**may include references**)	p. 3
Patient Information	5a	De-identified patient specific information.	p. 3
	5b	Primary concerns and symptoms of the patient.	pp. 3-4
	5c	Medical, family, and psycho-social history including relevant genetic information	pp. 3-4
	5d	Relevant past interventions with outcomes	p. 4
Clinical Findings	6	Describe significant physical examination (PE) and important clinical findings.	p. 3
Timeline	7	Historical and current information from this episode of care organized as a timeline	p. 6, Table 1
Diagnostic Assessment	8a	Diagnostic testing (such as PE, laboratory testing, imaging, surveys).	pp. 4-5
	8b	Diagnostic challenges (such as access to testing, financial, or cultural)	pp. 4-5
	8c	Diagnosis (including other diagnoses considered)	pp. 5, 7-10
	8d	Prognosis (such as staging in oncology) where applicable	outcome p. 6
Therapeutic Intervention	9a	Types of therapeutic intervention (such as pharmacologic, surgical, preventive, self-care)	pp. 4-5
	9b	Administration of therapeutic intervention (such as dosage, strength, duration)	pp. 4-5
	9c	Changes in therapeutic intervention (with rationale)	pp. 4-5, 10-11
Follow-up and Outcomes	10a	Clinician and patient-assessed outcomes (if available)	pp. 5-6
	10b	Important follow-up diagnostic and other test results	pp. 5-6
	10c	Intervention adherence and tolerability (How was this assessed?)	p. 5
	10d	Adverse and unanticipated events	p. 5
Discussion	11a	A scientific discussion of the strengths AND limitations associated with this case report	p. 11
	11b	Discussion of the relevant medical literature **with references**.	pp. 7-11
	11c	The scientific rationale for any conclusions (including assessment of possible causes)	pp. 9-11
	11d	The primary "take-away" lessons of this case report (without references) in a one paragraph conclusion	p. 11
Patient Perspective	12	The patient should share their perspective in one to two paragraphs on the treatment(s) they received	p. 6
Informed Consent	13	Did the patient give informed consent? Please provide if requested	Yes ☒ No ☐
